# The role of tRNA synthetases in neurological and neuromuscular disorders

**DOI:** 10.1002/1873-3468.12962

**Published:** 2018-02-01

**Authors:** Veronika Boczonadi, Matthew J. Jennings, Rita Horvath

**Affiliations:** ^1^ Wellcome Centre for Mitochondrial Research Institute of Genetic Medicine Newcastle University Newcastle upon Tyne UK

**Keywords:** aminoacyl‐tRNA synthetases, cytosolic and mitochondrial translation, Charcot‐Marie‐Tooth disease

## Abstract

Aminoacyl‐tRNA synthetases (ARSs) are ubiquitously expressed enzymes responsible for charging tRNAs with their cognate amino acids, therefore essential for the first step in protein synthesis. Although the majority of protein synthesis happens in the cytosol, an additional translation apparatus is required to translate the 13 mitochondrial DNA‐encoded proteins important for oxidative phosphorylation. Most ARS genes in these cellular compartments are distinct, but two genes are common, encoding aminoacyl‐tRNA synthetases of glycine (*GARS*) and lysine (*KARS*) in both mitochondria and the cytosol. Mutations in the majority of the 37 nuclear‐encoded human ARS genes have been linked to a variety of recessive and dominant tissue‐specific disorders. Current data indicate that impaired enzyme function could explain the pathogenicity, however not all pathogenic ARSs mutations result in deficient catalytic function; thus, the consequences of mutations may arise from other molecular mechanisms. The peripheral nerves are frequently affected, as illustrated by the high number of mutations in cytosolic and bifunctional tRNA synthetases causing Charcot–Marie–Tooth disease (CMT). Here we provide insights on the pathomechanisms of CMT‐causing tRNA synthetases with specific focus on the two bifunctional tRNA synthetases (*GARS*,* KARS*).

## Abbreviations

**ARSAL**, Autosomal Recessive Spastic Ataxia with Leukoencephalopathy

**ARSs**, Aminoacyl‐tRNA synthetases

**CMT**, Charcot–Marie–Tooth disease

**GARS**, glycyl‐ARS

**MLASA**, mitochondrial myopathy, lactic acidosis and sideroblastic anaemia

**MSC**, multi‐tRNA synthetase complex

**NMJs**, neuromuscular junctions

**VEGF**, vascular endothelial growth factor

Aminoacyl‐tRNA synthetase proteins (ARS) are a family of nuclear‐encoded enzymes that ensure correct translation of the genetic code by conjugating each of the 20 amino acids to their cognate tRNA molecule 1, 2, 3. This aminoacylation reaction provides the substrate for the protein translation process. There are two groups of ARS enzymes: the cytosolic ARS, which are responsible for supplying aminoacyl‐tRNA conjugates for general protein translation; mitochondrial ARSs, which are imported into the mitochondrial matrix and charge amino acids to their mitochondrial genome‐encoded tRNA molecules (mt‐tRNA). For most amino acids, there are dedicated isoforms encoded in the genome for each of these compartments, but in the case of glycyl‐ARS (GARS) and lysyl‐ARS (KARS), the proteins are bifunctional and localised to both the cytosol and the mitochondria 4. Furthermore, no mitochondrial glutamyl‐tRNA synthetase (QARS) has been identified; Q‐tRNA is instead suggested to be formed by postconjugation modification of glutamic acid 5.

## Mitochondrial tRNA synthetases (*ARS2* genes)

**Figure 1:**
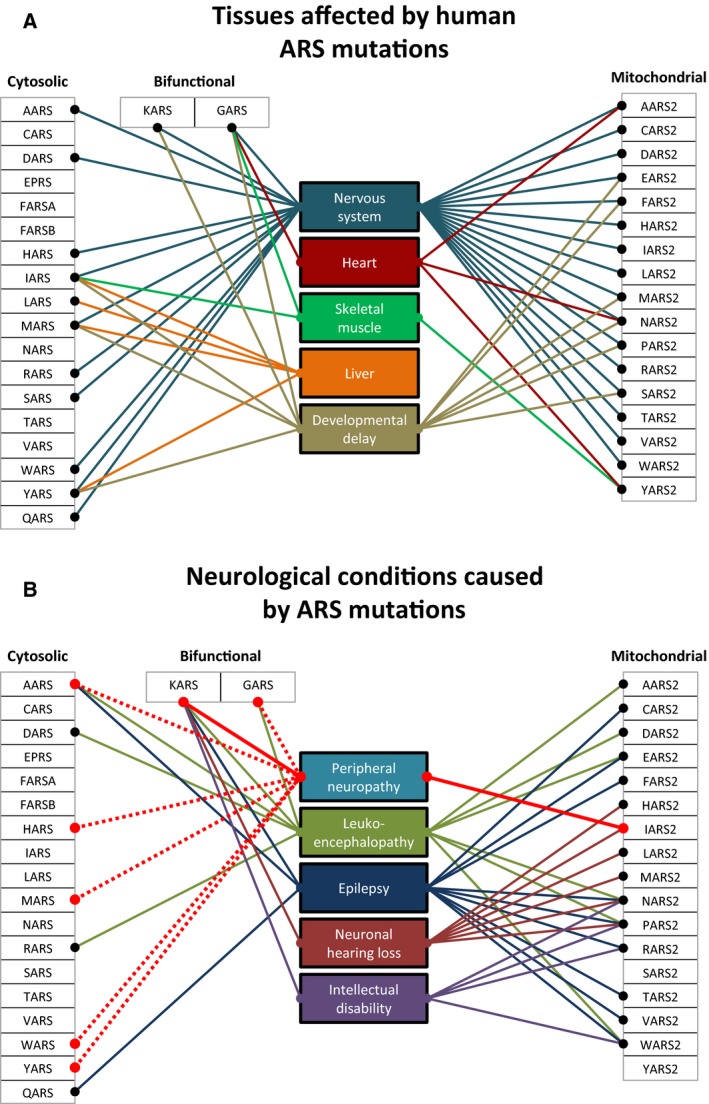
Clinical variability of diseases caused by ARSs mutations. (A) Tissues commonly affected by mutations in cytosolic, bifunctional and mitochondrial ARS genes. (B) Common neurological presentations reported in cytosolic, bifunctional and mitochondrial ARS genes, with peripheral neuropathy highlighted. Solid line indicates dominant mode of inheritance, dashed line indicates recessive mode of inheritance. References: *AARS* 33, 48, 50, 96, *DARS* 97,*HARS* 27, *IARS* 37, 40 *MARS* 45, 98, *RARS* 99, S*ARS* 18, W*ARS* 30, *ARS* 28, 39, *QARS* 34, *GARS* 7, 29, 71, *KARS* 38, *AARS2* 10, 67, *CARS2* 12, *DARS2* 8, *EARS2* 9, *FARS2* 13, 100, *HARS2* 21, *IARS2* 101, 102, *LARS2* 22, *MARS2* 11, 19, *NARS2* 20, 103, 104, *PARS2* 14, 103, *RARS2* 17, 105, S*ARS* *2* 24, *TARS2* 15, *VARS2* 15, 16, *WARS2* 18, 106, *YARS2* 23, 107.

All mt‐ARSs are synthesised in the cytosol; addressed to, and imported into, the mitochondria due to the presence of an N‐terminal presequence (mitochondrial targeting sequence), which is cleaved upon entry to the mitochondria 6. Pathogenic variants of mt‐ARSs show a variety of phenotypes involving tissues with high energy demand. Despite their crucial housekeeping function and ubiquitous expression, mutations in mt‐ARSs have been implicated in a variety of paediatric and adult onset human neurological disorders of the brain, spinal cord and motor neurons in addition to disorders predominantly affecting other tissues manifesting as cardiomyopathy, myopathy, sensorineural hearing loss and endocrine symptoms 1, 2, 3, 7. A large number of autosomal recessive disorders specifically affect the brain, and result in lesions of certain neuronal cell types. The most typical clinical presentations are leukoencephalopathy with brainstem and spinal cord involvement and high lactate (LBSL) due to *DARS2* mutations 8, leukoencephalopathy with thalamus and brainstem involvement and high lactate (LTBL) caused by *EARS2* mutations 9, but other mt‐ARS mutations may also cause white matter lesions. Mutations within the *AARS2* gene result in two different phenotypes: late onset ovarian failure and leukodystrophy and infantile mitochondrial cardiomyopathy 10. *MARS2* mutations have been previously linked to Autosomal Recessive Spastic Ataxia with Leukoencephalopathy (ARSAL) 11, presentations similar to that observed in Alpers–Huttenlocher syndrome have been reported in patients with mutations in *CARS2* 12, *FARS2* 13 *, PARS2* 14, *TARS2* 15 *, VARS2* 16 NARS2 16, *RARS2* 17 and *WARS2* 18. These diseases display a broad clinical spectrum and may be further complicated with other symptoms such as developmental delay (*NARS2, PARS2*), pontocerebellar hypoplasia (*RARS2*), visual impairment (*FARS2*) psychomotor delay (*TARS2*) and microcephaly (*VARS2*). Other ARS2 diseases show characteristic tissue distribution, isolated sensorineural hearing loss (*MARS2* 19, *NARS2* 20), hearing loss with premature ovarian failure (Perrault syndrome, *HARS2* 21 and *LARS2* 22), mitochondrial myopathy, lactic acidosis and sideroblastic anaemia (MLASA syndrome (*YARS2* 23), or hyperuricaemia, pulmonary hypertension renal failure and alkalosis (HUPRA syndrome, *SARS2* 24). To date, no ARS2 mutations have been reported in human disease with an autosomal dominant mode of inheritance, all mutations are either homozygous or compound heterozygous (Fig. 1).

## Bifunctional and cytosolic tRNA synthetases

While autosomal dominant pathogenic variants of the cytosolic ARSs were originally reported in peripheral neuropathies, newly emerging data show a spectrum of recessive disorders. Autosomal dominant neuropathies have been associated with mutations in *AARS* 25, 26, *HARS* 27, *YARS* 28, *GARS* 29 and *WARS* 30. Autosomal recessive ARS mutations have been recently reported in a range of conditions often affecting the central nervous system (microcephaly *AARS*,* QARS* 31, 32), epileptic encephalopathy (*AARS*,* QARS* 31, 32, 33, 34), sensorineural hearing loss (*HARS*,* KARS* 35, 36), developmental delay (*IARS*,* KARS*,* QARS*,* YARS* 34, 37, 38, 39), or causing liver dysfunction (*IARS*,* MARS*,* YARS* 39, 40, 41) and lung disease (*MARS* 39, 41). The large variability of the clinical presentations caused by mutations even in a single ARS gene is remarkable and needs further investigations (Fig. 1).

## Charcot–Marie–Tooth disease caused by defects of ARS mutations

Charcot–Marie–Tooth disease (CMT) was the first human disorder to be linked to ARSs mutations. CMT is the most common inherited neurological disease in European populations, with an estimated prevalence of around 1–4 per 10 000 individuals 42, 43, and is characterised by symmetric atrophy and weakness in the distal muscles associated with sensory impairment caused by progressive degeneration of the peripheral nerves. CMT is broadly divided into two forms: demyelinating (CMT1) primarily affecting the myelin sheaths of peripheral neurons or axonal form (CMT2) primarily affecting the axons. These can be differentiated based on the electrophysiological and pathological presentation, however intermediate phenotypes exist 44. Five of the 20 genes encoding cytosolic ARSs (*AARS, GARS, WARS, YARS* and *HARS*) have been reported to cause dominant inherited CMT, with the number expected to increase, and remarkably, no other dominantly inherited disease has been linked with mutations (Fig. 1). Furthermore, two additional ARS genes have also been associated with CMT. Heterozygous missense mutations in *MARS* have been found in two families causing CMT2U 45, 46 and peripheral neuropathy was reported in two patients with compound heterozygous mutations in *KARS* 38.

**Figure 2:**
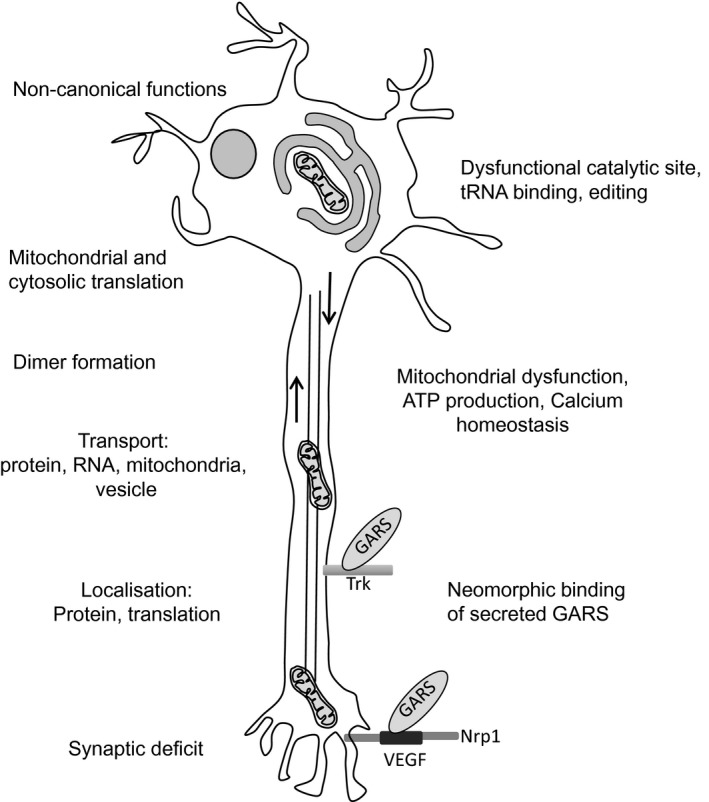
Possible pathological pathways of axonal degeneration in tRNA synthetase mutations.

Besides the fact that new mutations are continuously discovered, neither the cause of the selective vulnerability, nor the molecular mechanisms leading to the disease, are well understood. Currently, there are no disease modifying therapies for CMT and clinical care focuses on managing symptoms with physical therapy and orthopaedic surgery. The similarity of clinical presentation resulting from dominant inherited ARS mutations implies a shared mechanism of disease, though no mechanism so far has been proven. The major mechanisms proposed consist of: reduced aminoacylation activity, altered dimerisation or localisation, gain‐of‐function pathogenic interactions and loss of noncanonical function (Fig. 2). We examine the evidence supporting, contradicting and lacking for each mechanism.

## Evidence of impaired catalytic function

The central housekeeping role of ARS proteins in cellular physiology implies that defects in their function would cause cellular dysfunction. Reduction in both the aminoacylation reaction rate and cognate accuracy of the enzyme present possible pathogenic mechanisms. ARS proteins act via a two‐step process to facilitate tRNA‐amino acid conjugation and the activity of mutant ARS enzymes can be determined *in vitro* using recombinant human protein 47. These enzymatic assays have generally found pathogenic variants of cytosolic ARS to cause at least some degree of reduced enzymatic activity, with the notable exception of the *YARS* p.Glu196Lys variant (Table 1). Yeast complementation studies on the translational function of the mutant ARS and constructs using CMT‐associated variants have been shown to cause either lethality or reduced growth in most, but not all, cytosolic ARS enzymes (Table 1). Although there is strong evidence of impaired catalytic function in CMT‐associated cytosolic ARS variants, the potential mechanism is less well characterised; with possibilities including dysfunction of the catalytic site, abnormal protein dimer formation and altered localisation.

**Table 1:** Functional evidence on pathogenicity of CMT‐causing tRNA synthetase mutations. CMT, Charcot–Marie–Tooth disease; dHMN, distal hereditary motor neuropathy; AARS, alanyl‐tRNA synthetase; YARS, tyrosine‐tRNA synthetase; HARS, histidyl‐tRNA synthetase; MARS, methionyl‐tRNA synthetase; WARS, tryptophanyl‐tRNA synthetase; GARS, glycyl‐tRNA synthetase; KARS, lysyl‐tRNA synthetase; NRP1, Neuropilin‐1 precursor; VEGF, Vascular endothelial growth factor; TrkA/B/C, tropomyosin receptor kinase A/B/C

	Experimental method	Effect of mutations
Cytosolic tRNA synthetase		
*AARS*	Recombinant enzyme activity:	Reduced: p.Asn71Tyr 50; p.Arg329His 50;
	Yeast growth:	Lethal: p.Asn71Tyr 50; Gly102Arg 108 p.Arg329His 50
	Mouse:	Purkinje cells loss, ataxia, no effect on NMJ morphology: Ala734Glu 109 (mutation in mouse *Aars*)
*HARS*	Yeast growth:	Lethal: p.Thr132Ile 27; p.Pro134His 27; p.Arg137Gln 52; p.Asp364Tyr 27 Reduced: p.Asp175Glu 27
	*Caenorhabditis elegans*:	Motor neuron toxicity: p.Arg137Gln (targeted to 4‐aminobutyrate neurons) 52
*MARS*	Yeast growth:	Lethal: p.Arg618Cys (mutation has incomplete penetrance) 45 p.Pro800Thr lacks functional evidence
*WARS*	Recombinant enzyme activity:	Abnormal: His257Arg 30
	Yeast growth:	Abnormal: Hist257Arg 30
*YARS*	Recombinant enzyme activity:	Reduced: p.Gly41Arg 110; p.153‐156delValLysGlnVal 110 Conflicting (reduced and normal): p.Glu196Lys 28, 110
	Yeast growth:	Lethal: p.Gly41Arg 28 Reduced: p.Glu196Gln 51; p.Glu196Lys (yeast orthologue, human is normal) 28
	Human N2a neuroblastoma cells:	Reduced axonal distribution: p.Gly41Arg 28,
Bifunctional tRNA synthetase		
*GARS*	Recombinant enzyme activity:	Reduced: p.Ala57Val (minor reduction) 61; p.Leu129Pro 111; p.Asp146Asn 61; p.Ser211Phe 61; p.Gly240Arg 61; p.Pro244Leu 61; p.Ile280Phe 61; p.His418Arg 61; p.Gly526Arg 61; p.Gly598Ala 61 Normal: p.Glu71Gly 54; p.Asp500Asn 54; p.Ser581Leu 54
	Yeast growth:	Lethal: p.Pro244Leu; p.His418Arg 111; p.Gly526Arg 111; Reduced: p.Leu129Pro 111; p.Asp146Asn 61 Normal: p.Glu71Gly 111; p.Gly240Arg 60, 111; p.Ile280Phe 61; p.Gly598Ala 60
	*Drosophila*:	Abnormal dendritic morphology, altered mitochondrial translation, and progressive NMJ degeneration: p.Glu71Gly 89; p.Gly240Arg 89, 112; p.Gly526Arg 89; p.Pro234Lys,Tyr 112
	Mouse:	Secreted mutant GARS binds to NRP1, interfering with VEGF NMJ dysfunction, aberrant binding to TrkA, TrkB, and TrkC, receptors in sensory neurons: p.Pro278Lys 75, 76; p.Cys201Arg 75, 76
*KARS*	Recombinant enzyme activity:	Reduced: p.Leu133His 38
	Yeast growth:	Lethal: p.Tyr173Serfs*7 38 Normal: p.Leu133His 38

By mapping ARS mutations onto the 3D protein structures determined by X‐ray crystallography, it is possible to determine whether they are clustered around particular functional sites. The p.Asp71Tyr and p.Gly102Arg *AARS* mutations are predicted to lie within the catalytic domain, whereas the common p,Arg329His mutation is predicted to be at the tRNA‐binding domain and the p.Asp893Asn and p.Glu688Gly mutations are reported within the editing domain 48, 49, 50. Moving to *YARS* and *HARS*, most reported mutations are predicted to lie on the catalytic domain, though importantly, unlike *AARS*, these proteins do not contain editing domains 28, 46, 51, 52, 53. Though no crystal structure has been determined for MARS, computational prediction maps one mutation, p.Arg6128Cys, between the catalytic and tRNA‐binding domains, and another mutation, p.Pro800Thr, to the tRNA‐binding domain 45, 46, while the recently reported *WARS* mutation, p.His257Arg, is in the catalytic domain 30. The diversity of mutations between the different major domains reported for cytosolic ARS rules out dysfunction at either the catalytic site or in tRNA‐binding as the sole mode of pathogenicity. Furthermore, the lack of clustering within any domain suggests impairment of canonical domain function may not be relevant to the disease pathophysiology, with secondary roles of the affected residues potentially being important.

Questions remain as to how the reduction in catalytic capacity in dominant mutations causes peripheral neuropathy specifically, without affecting other tissues with high demand for protein synthesis. A possibility is that similar biochemical impairment is seen as in recessive phenotypes but milder due to the presence of some wild‐type protein and therefore specifically effecting the unusual proteomic demands of peripheral nerves. Alternatively, dimerisation, localisation or more complex elements specific for peripheral nerves such as neuromuscular junction, neuronal receptors (neuropilin 1) may be involved in conjunction with the catalytic deficit to cause peripheral nerve‐specific presentation. The question about aminoacylation deficiency and disease causality is controversial and still open in the literature, although we think that it may be causative at least in some mutations. However, other pathological mechanisms may have an additive effect and for certain mutations aminoacylation is not altered and different factors may explain the disease phenotype.

## Dimerisation

All CMT‐associated ARS enzymes (AARS, GARS, WARS, YARS, HARS) catalytically function as dimers, and disruption to the dimer functionality has been suggested as a mechanism of pathogenicity in ARS‐related CMT. Clustering of mutations of GARS has been reported around the homodimer interface, and mutations affecting the dimer interface have been shown to cause subtle conformational alterations to mutant wild‐type dimers 54, 55. In addition, Dewan *et al*. 56 demonstrated that due to a H7 helix located in motif 1 of human KARS (member of the multi‐synthetase complex), it is also capable of dimerisation. The effects of mutations on dimerisation have not been reported for either AARS or WARS, and some YARS mutations have been found to have no effect on monomer–dimer equilibrium 57, 58, 59. A role of dimerisation presents several mechanistic possibilities, namely that (a) CMT‐associated variants cannot form dimers and are therefore inactive, (b) CMT‐associated variants form unstable dimers which are subsequently either aggregated or degraded or that (c) CMT‐associated variants do form stable dimers which are either deficient in aminoacylation activity, deficient in other noncanonical functions or have a toxic gain‐of‐function.

Addressing the first possibility, several *GARS* and *YARS* variants have been shown to have binding affinities equivalent to, or exceeding that of, the wild‐type protein 54, 57, thus ruling out decreased dimerisation. Concerning dimer stability, structural studies of CMT‐associated YARS variants show both increased and decreased protein stability, suggesting protein instability and aggregation *per se* is not a common mechanism. Animal studies of mutant and wild‐type *YARS* and *GARS* have shown equivalent expression of the protein in the cell body 54, 60, 61, and furthermore, specific quantification of the transfected mutant GARS is equivalent at the cell body to wild‐type 54, showing that the expression level of the protein is unaffected and therefore degradation is not a factor. It must therefore be asked: what evidence exists for the role of stable toxic ARS dimers? *In vitro* enzymatic assays have already shown that although aminoacylation is often affected in mutant homodimers, it is not a common feature of all CMT‐associated mutations and therefore, considering the dominant mode of inheritance, it should be examined whether the aminoacylation activity of tRNA heterodimers may have a role. A study, looking at CMT‐associated mutations in zebrafish showed that overexpression of the homologue of the nondimerising p.Thr130Lys mutation reduced NMJ toxicity of the CMT‐associated p.Gly526Arg mutation when alongside normal expression of wild‐type *gars*, suggesting that dimerisation of the mutant protein with the wild‐type is necessary for pathogenicity 62. Supporting the potential for a stable dimer engaging in toxic neomorphic binding, a recent study examined the structural and catalytic properties of three CMT‐associated *YARS* variants and showed that, while other properties such as conformational alteration of dimer structure were not common, exposure of an internal site on the dimers was, and this facilitates increased binding to TRIM28 57. How this or other neomorphic‐binding partners are involved in the pathogenesis of remains to be elucidated, but it provides a principle along which variants in other genes could be examined.

## Cellular localisation

Alteration to the cellular distribution of cytosolic enzymes presents another pathogenic mechanism. If mutations disrupt the distribution of ARS proteins within the cell, and cause a deficit of protein activity in the axons of peripheral neurons, which are often much longer than axons of the central nervous system, this may plausibly explain why mutations cause length‐dependent pathology in the long axons. However, the few studies that have investigated distribution both *in vitro* and *in vivo* have shown mixed results in alterations resulting from CMT‐causing mutations. In the case of *YARS,* a clustering of expression can be observed around neuronal projections, which was abolished in N2a cells transfected with CMT‐causing *YARS* mutations (p.Gly45Arg, p.Gln196Lys) 28. A *HARS* mutation, p.Arg137G 52, modelled in *Caenorhabditis elegans* has shown a normal pattern of axonal expression alongside morphological abnormalities and three different *YARS* variants (p.Gly41Arg, p.153‐156delValLysGlnVal, p.Glu196Lys) modelled in *Drosophila* showed no change in axonal localisation. Also the distribution of the mutant Gars in a CMT2D mouse model indicated unaltered localisation in the sciatic nerve fibres 41. Therefore, despite evidence of altered localisation in some ARS mutations, it is currently unknown whether it is a common mechanism of disease.

## Noncanonical functions

The lack of clear mechanistic evidence supporting deficits in aminoacylation (catalytic function) has led the suggestion that cytosolic ARS disease may relate to entirely different ‘noncanonical’ ARS functions. This could include the loss of a noncanonical physiological ARS function, or alternatively can be a gain of pathological function. During the evolutionary process, cytosolic ARS proteins, or complexes formed from them, have accrued additional functions to their core ‘canonical’ role in aminoacylating tRNA molecules with roles as diverse as mRNA splicing 63, modulation of angiogenesis 64 as well as roles in injury repair in the peripheral nervous system 65, 66. Whether disease‐associated mutations in cytosolic ARSs cause disruption to a noncanonical function in the peripheral nervous system, remains to be confirmed, and if any alterations are found it is important to show that this is replicated across different mutations and critically across the disease‐associated genes, which are not known to share many noncanonical functions. Investigation of loss of noncanonical function is further complicated as known functions are often not present in lower organisms, therefore limiting the relevance of more simple biological models such as yeast or *C. elegans*.

## Neuromuscular diseases caused by defects of bifunctional ARS mutations

Reported mutations in bifunctional enzymes, GARS and KARS show either recessive or dominant inheritance, with strikingly different tissue specific clinical manifestations. Dominant *GARS* mutations have been identified in patients with axonal peripheral neuropathy (CMT2D or distal spinal muscular atrophy type: dSMA‐V) 29, 55 and recessive mutations were reported with patients with predominant cardiomyopathy leading to death in an infant or cardiomyopathy and mitochondrial myopathy with exercise intolerance 7, 67, 68. Mutations in *KARS* have been so far associated with various phenotypes: autosomal recessive CMT 38, nonsyndromic hearing loss 36, childhood‐onset visual impairment with progressive microcephaly with combined mitochondrial respiratory chain defect 68, and a severe cardiomyopathy associated with myopathy, intellectual disability and lactic acidosis 69, and recently with early‐onset, profound sensorineural hearing loss and leukoencephalopathy 70, making clear genotype–phenotype correlations difficult for *KARS*.

Despite of many efforts it is not clear how variants of bifunctional ARSs impact the mitochondrial function and whether or not the mitochondrial impairment is solely recessive, similar to mutations in other nuclear‐encoded genes affecting mitochondrial translation, while neuropathy is caused by the defect of another, probably cytosolic, function.

With the exceptions of one patient with recessive *KARS* mutations developed CMT, recessive *GARS* or *KARS* mutations are not associated with peripheral neuropathy, rather with prominent heart and skeletal muscle dysfunction. As the majority of patients with recessive *GARS* and *KARS* mutations are children, it is possible that neuropathy will develop in a later stage; however, no reliable clinical data support this hypothesis. There is only limited clinical information available on parents of children with recessive disease, who carry heterozygous *GARS* or *KARS* mutations. In one case, the father presented with mild neuropathy detected on electrophysiology, but the mother showed no clinical or electrophysiological alterations 71. These data suggest that the mechanism of disease is different for dominant and recessive mutations. It is possible that recessive mutations have a detrimental effect on mitochondrial translation only in homozygous state or in combination with another heterozygous pathogenic mutation, leading to tissue‐specific manifestations in the heart, skeletal muscle, brain or inner ears potentially by loss‐of‐function, whereas for dominant mutations, another mechanism leads to peripheral nerve lesion, one similar to other cytosolic CMT‐causing ARS mutations (see above) (Fig. 2, Table 1).

## Gain‐of‐function through neomorphic binding

Among various ARSs, the secretion of wild‐type KARS (colon cancer cells, macrophages) and GARS (immune cells, mouse motor neurons and differentiated myotubes) to extracellular space via exosomes has been confirmed by several studies 72, 73. Since this observation, it has been investigated whether pathogenic *GARS* mutations introduce abnormal conformational changes leading to aberrant protein interactions. Supporting a toxic gain‐of‐function, recent findings in mice carrying pathogenic *Gars* mutations show that the mutant Gars protein acquires a neomorphic‐binding activity that directly antagonises an essential signalling pathway for motor neuron survival 73. CMT‐causing mutations alter the conformation of Gars, enabling it to bind the neuropilin 1 (Nrp1) receptor. This aberrant interaction competitively interferes with the binding of the cognate ligand vascular endothelial growth factor (VEGF) to Nrp1. Genetic reduction of Nrp1 in mice worsens the neuropathy, whereas enhanced expression of VEGF improves motor function. These findings link the selective neuronal pathology to the neomorphic‐binding activity of mutant GARS that antagonises the VEGF–Nrp1 interaction, and indicate that the VEGF–Nrp1 signalling axis is an actionable target for treating CMT2D. While VEGF–NRP1 signalling has been implicated to play a role in the nervous system 74 supporting its role in neuronal migration and axonal guidance, this pathway does not fully explain the late onset disease progression. This pathway was also linked to developmental cardiovascular defects and vascular homeostasis which was not observed in Gars mouse models 75. Furthermore, abnormal interference of mutant GARS with Trk receptors has been shown to have a role in the development of sensory neurons 76. Exploration of the interacting partners of several mutant ARS variants may shed light on a common pathological mechanism explaining the neuromuscular phenotype.

## Mitochondrial function

Several genetic forms of CMT are caused by mutations in proteins affecting mitochondrial function such as mitofusin 2 (MFN2), ganglioside‐induced differentiation‐associated‐protein 1 (GDAP1), heat shock protein beta 8 (HSPB8), and heat shock protein beta 1 (HSPB1) 77, suggesting that mitochondria are important in motor neurons. As mutations in other cytosolic ARSs cause similar neuropathies, it is possible that combination of several mechanisms including both cytoplasmic and mitochondrial function might play a role in the disease phenotype, or a so far unknown noncanonical function specifically targeting peripheral nerves explains why peripheral neuropathy is seen in isolation.

Investigation of mitochondrial function of bifunctional ARS has been reported in several studies. A mitochondrial isoform of KARS have been reported to interact with SOD1 in the mitochondria of the nervous system 78, which was suggested to contribute to mitochondrial dysfunction in ALS. More extensive studies showed loss‐of‐function effect of *GARS* mutations in *Drosophila*, which leads to progressive defects of dendritic morphology due to altered mitochondrial translation 79. Spaulding and her colleges also found an interesting link by identifying significantly fewer mitochondria at nerve terminals in two different mouse models of *GARS* mutations 80. This observation highlights that mitochondrial function may be altered in *GARS* mutations and a variety of mechanisms may underlie the mitochondrial dysfunction, including deficits in ATP production, failure of axonal transport, or changes in intracellular calcium dynamics. Keeping in line with this, antioxidants including ascorbic acid and forms of glutathione were reduced in mutant Gars^*Nmf249/+*^ mice spinal cord and sciatic nerve, suggesting changes in oxidative pathways 81. Therefore, mitochondrial dysfunction, whether primary or secondary, can be suggested as contributing factor and further studies on animal models in combination with patient‐derived motor neurons are warranted. Our preliminary data in *GARS*‐related neuropathy suggest that mitochondrial dysfunction involving very specific pathways is essential for the motor nerve dysfunction (unpublished data).

## Synaptic dysfunction

As neurons and muscle fibres are highly metabolically active, it is rational to hypothesise that the neuromuscular junctions (NMJs) are affected by even minor mitochondrial dysfunction. Recent data showed synaptic maturation abnormalities with specific, progressive NMJ degeneration in *Drosophila* where ubiquitous mutant gars was expressed 82. In addition to this, abnormal axonal transport was also suggested as potential disease mechanisms, which have been reported in a range of CMT2 models 60. To further strengthen this theory, both Gars mutant mouse models show muscle atrophy associated with compromised development of the NMJ prior to synaptic degeneration and highlight the neuromuscular synapse as an important site of early, selective pathology in CMT2D mice 83, 84.

## Abnormal axonal translation

The housekeeping function of the ARSs is to catalyse the aminoacylation of tRNA with their cognate amino acid, which is the first step of protein synthesis. Data from *in vitro* aminoacylation assay and yeast complementation studies showed that some CMT mutations do not affect the enzymatic activity, indicating that loss of aminoacylation activity alone is not required to cause peripheral neuropathy 85. Furthermore, in both CMT2D mouse models (Gars^*Nmf249/+*^ and Gars^*C201R/+*^ mice), mutations in Gars do not reduce tRNA^Gly^ aminoacylation activity 54, 83, 86, 87. On the other hand, the long axons of the peripheral nervous system could be particularly sensitive to defective protein translation. This idea, that mutant ARSs could affect protein translation within the axons, was investigated in a *Drosophila* model using a new method based on noncanonical amino acid tagging, which allows to cell‐type‐specifically monitor translation *in vivo* 88, 89. Direct evaluation of protein translation rates in sensory and motor neurons expressing CMT‐associated mutant GARS (both cytoplasmic and mitochondrial forms) and YARS showed global translational slowdown which was not attributed to the aminoacylation activity. It is therefore possible that defective protein synthesis locally in the axons act as a common pathogenic mechanism underlying mutant tRNA synthetase‐associated CMT 89. With the focus on bifunctional ARSs more in‐depth study required to investigate how the mutant GARS and KARS affect cytosolic and mitochondrial protein synthesis in isolation and how the variants influence the interaction between two translational systems.

## Noncanonical functions of bifunctional ARSs

It is well documented that several cytoplasmic ARSs acquired unique signal mediators during evolution, which facilitate numerous noncanonical biological processes 90. Among the various functions for ARSs includes inflammation, transcriptional regulation, translational regulation, apoptosis, rRNA transcription, angiogenesis, cell signalling, autoimmune response, tRNA maturation and mitochondrial RNA splicing 91. Also, several cytosolic and bifunctional ARSs have been linked to biological processes besides protein translation. Detection of GARS in the serum of normal human subjects and mouse 72 as well as in patients with cancer 92 indicates a role in immune defence system, and its therapeutic potential against tumorigenesis has been suggested 72. Other studies showed a chaperone‐like function for GARS whereby GARS interacts with ubiquitin‐like proteins and facilitates neddilation, which critically regulates cell cycle progression by degrading key regulators of the cell cycle and hence play a crucial role in selective protein degradation 93.

The cytosolic form of KARS is part of the multi‐tRNA synthetase complex (MSC) and involved in the housekeeping role; however, N‐terminal cleavage of KARS by caspase‐8 and an interaction with syntenin through its C‐terminal end leads to dissociation from the MSC complex and translocation to the plasma membrane where it associates with and stabilises a 67‐kDa laminin receptor (p67LR) 94. A study on HCT116 colon cancer cells showed that KARS localised to the plasma membrane is involved in cell–cell adhesion and more importantly suppression of KARS leads to impaired migratory abilities 95. Migration defects in CMT‐associated mutations may be involved in peripheral nerve lesions. Our group identified delayed cellular migration in patient‐derived cells carrying pathogenic dominant *GARS* mutations (unpublished data).

Recently, new investigations using a metabolomics analysis in a mouse model of CMT2D, (*Gars* ^*Nmf249/+*^) attempted to identify changes in metabolite abundance that may be indicative of the pathophysiology 81. Despite the fact that the metabolomics analysis of spinal cord from a CMT2D mouse model revealed distinct metabolite fingerprints, associated with the disease (ascorbic acid, carnitine, glycine), none of the studied candidates proved to be specific to the disease development or were confirmed in CMT patient samples. Still the potential promise of metabolite profiling to understand disease mechanisms cannot be disregarded.

## Conclusions and perspectives

In this review, we summarised the research on the pathomechanisms of ARS mutations causing peripheral neuropathy; however, what remains particularly unclear is the cause of the high degree of tissue specificity. Some excellent *in vivo* (mice, *Drosophila*) and *in vitro* (human fibroblasts, iPSCs, neuronal cells) model systems have been developed to study ARSs in different tissues, which will hopefully provide further insights into the disease mechanism. Various noncanonical functions of ARSs have become increasingly interesting, and by being secreted could have widespread effects or could act as potential biomarkers. To date, there is no good biomarker available to study the progression of neuropathy in these slowly progressive diseases. Understanding why peripheral nerves are predominantly affected will open potential therapeutic targets for a larger group of CMT patients; however, further research is still needed.
